# Subtyping Social Determinants of Health in the "All of Us" Program: Network Analysis and Visualization Study

**DOI:** 10.2196/48775

**Published:** 2025-02-11

**Authors:** Suresh K Bhavnani, Weibin Zhang, Daniel Bao, Mukaila Raji, Veronica Ajewole, Rodney Hunter, Yong-Fang Kuo, Susanne Schmidt, Monique R Pappadis, Elise Smith, Alex Bokov, Timothy Reistetter, Shyam Visweswaran, Brian Downer

**Affiliations:** 1 School of Public and Population Health Department of Biostatistics & Data Science University of Texas Medical Branch Galveston, TX United States; 2 Department of Radiology Houston Methodist Houston, TX United States; 3 Department of Internal Medicine Division of Geriatrics Medicine University of Texas Medical Branch Galveston, TX United States; 4 College of Pharmacy and Health Sciences Department of Pharmacy Practice Texas Southern University Houston, TX United States; 5 Department of Population Health Sciences University of Texas Health San Antonio San Antonio, TX United States; 6 School of Public and Population Health Department of Population Health & Health Disparities University of Texas Medical Branch Galveston, TX United States; 7 School of Public and Population Health Department of Bioethics & Health Humanities University of Texas Medical Branch Galveston, TX United States; 8 School of Health Professions Department of Occupational Therapy University of Texas Health San Antonio San Antonio, TX United States; 9 Department of Biomedical Informatics University of Pittsburgh Pittsburgh, PA United States

**Keywords:** social determinants of health, All of Us, bipartite networks, financial resources, health care, health outcomes, precision medicine, decision support, health industry, clinical implications, machine learning methods

## Abstract

**Background:**

Social determinants of health (SDoH), such as financial resources and housing stability, account for between 30% and 55% of people’s health outcomes. While many studies have identified strong associations between specific SDoH and health outcomes, little is known about how SDoH co-occur to form subtypes critical for designing targeted interventions. Such analysis has only now become possible through the *All of Us* program.

**Objective:**

This study aims to analyze the *All of Us* dataset for addressing two research questions: (1) What are the range of and responses to survey questions related to SDoH? and (2) How do SDoH co-occur to form subtypes, and what are their risks for adverse health outcomes?

**Methods:**

For question 1, an expert panel analyzed the range of and responses to SDoH questions across 6 surveys in the full *All of Us* dataset (N=372,397; version 6). For question 2, due to systematic missingness and uneven granularity of questions across the surveys, we selected all participants with valid and complete SDoH data and used inverse probability weighting to adjust their imbalance in demographics. Next, an expert panel grouped the SDoH questions into SDoH factors to enable more consistent granularity. To identify the subtypes, we used bipartite modularity maximization for identifying SDoH biclusters and measured their significance and replicability. Next, we measured their association with 3 outcomes (depression, delayed medical care, and emergency room visits in the last year). Finally, the expert panel inferred the subtype labels, potential mechanisms, and targeted interventions.

**Results:**

The question 1 analysis identified 110 SDoH questions across 4 surveys covering all 5 domains in *Healthy People 2030*. As the SDoH questions varied in granularity, they were categorized by an expert panel into 18 SDoH factors. The question 2 analysis (n=12,913; *d*=18) identified 4 biclusters with significant biclusteredness (*Q*=0.13; random-*Q*=0.11; *z*=7.5; *P*<.001) and significant replication (real Rand index=0.88; random Rand index=0.62; *P*<.001). Each subtype had significant associations with specific outcomes and had meaningful interpretations and potential targeted interventions. For example, the *Socioeconomic barriers* subtype included 6 SDoH factors (eg, *not employed* and *food insecurity*) and had a significantly higher odds ratio (4.2, 95% CI 3.5-5.1; *P*<.001) for depression when compared to other subtypes. The expert panel inferred implications of the results for designing interventions and health care policies based on SDoH subtypes.

**Conclusions:**

This study identified SDoH subtypes that had statistically significant biclusteredness and replicability, each of which had significant associations with specific adverse health outcomes and with translational implications for targeted SDoH interventions and health care policies. However, the high degree of systematic missingness requires repeating the analysis as the data become more complete by using our generalizable and scalable machine learning code available on the *All of Us* workbench.

## Introduction

### Background

Social determinants of health (SDoH), such as financial resources [1] and housing stability [2], account for between 30% and 55% of people’s health outcomes [3]. While many studies have identified strong associations between specific SDoH and health outcomes, most people experience multiple SDoH concurrently in their daily lives [4-8]. For example, limited access to education, unstable employment, and lack of access to health care tend to frequently co-occur across individuals, leading to long-term stress and depression [8]. Such complex interactions among multiple SDoH make it critical to analyze combinations of SDoH versus single factors. However, analysis of such co-occurrences and their risks of adverse health outcomes requires the integration of personal, clinical, social, and environmental information, critical for designing cost-effective and targeted interventions. Unfortunately, the lack of databases containing such multiple datatypes from the same individuals has resulted in a fragmented understanding of how SDoH co-occur and impact health, which is critical for designing targeted interventions.

The *All of Us* program [9-11] provides an unprecedented opportunity to address this fragmented view of SDoH. This program aims to collect data from multiple sources related to 1 million or more individuals, with a focus on populations that have been traditionally underrepresented in biomedical research. These data sources include electronic health records (EHRs), health surveys, whole-sequence genome data, physical measurements, and personal digital information. Critically, *All of Us* provides several survey modules containing a wide range of SDoH-related questions, which, in combination with other data sources, could transform our understanding of high-risk combinations of SDoH [9].

However, little is known about the range of and responses to SDoH questions in *All of Us* and how they co-occur to form subtypes, which are critical for designing targeted interventions. To address these gaps, we characterized 110 SDoH in *All of Us*, which guided the methods we used to analyze how they co-occur to form subtypes and their risk of adverse health outcomes. The results helped highlight the opportunities and challenges for conducting subtype analysis in *All of Us*, which integrates multiple datatypes through the use of scalable and generalizable machine learning methods aimed at designing targeted interventions.

### Models and Research Related to SDoH

The World Health Organization defines SDoH as the “non-medical factors that influence health outcomes” [3]. Specifically, these include the conditions in which people are born, grow, work, live, and age. Furthermore, such conditions are shaped by a wider set of forces, such as economic and social policies, and systems, such as discriminatory laws and structural racism.

**Figure 1:**
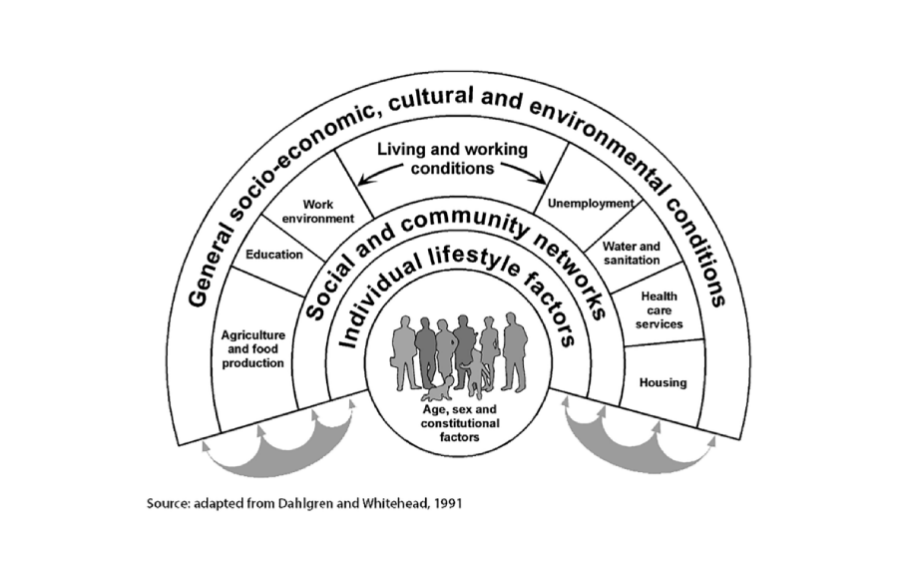
The Dahlgren-Whitehead conceptual model aimed at visually showing the interrelated layers of social determinant of health domains that influence health (reproduced from Dahlgren and Whitehead, 2021).

Several models have proposed the factors and mechanisms involved in SDoH [4,12]. These models were motivated by the concept of *social gradient* [13], an empirical phenomenon observed within and across nations [14,15] consistently showing that the lower an individual’s socioeconomic position, the worse their health. To help explain the factors underlying the social gradient, the model by Dahlgren and Whitehead [4,16] proposed several interconnected layers of social determinants that influence health. As shown in Figure 1 [4,16], the innermost layer comprises demographic and genetic factors, which are largely unmodifiable. In contrast, the outer layers are modifiable to different degrees, such as lifestyle (eg, exercise and smoking); social and community networks (eg, contact with supportive friends and family); living and working conditions (eg, access to health care and employment); and broader socioeconomic, cultural, and environmental conditions (eg, crime in the neighborhood). While this model was not intended to provide explicit testable hypotheses [4], the factors within each layer are expected to co-occur and impact each other in addition to responding to external forces such as systemic racism and capitalism when it is focused on financial profits at the expense of societal benefits.

These early SDoH models motivated numerous studies [17] that analyzed associations among specific SDoH (eg, immigration status and home density [7]), their association with health outcomes (eg, education and mortality [18]), and how they manifest within subpopulations (eg, patients with diabetes [19]). More recently, organizations such as the Centers for Disease Control and Prevention and *Healthy People 2030* (HP-30) have classified these empirical results into SDoH domains that roughly map to the Dahlgren-Whitehead model. For example, HP-30 organized SDoH empirical studies into five SDoH domains: (1) economic stability, (2) education access and quality, (3) health care access and quality, (4) neighborhood and built environment, and (5) social and community context. Furthermore, the PhenX program (which provides well-established measurement protocols for use in biomedical and translational research) has identified SDoH data collection protocols to enable more systematic data collection and analysis [20-22].

While the aforementioned findings and categorizations have greatly improved our understanding of SDoH and their impact on health, they have been mostly analyzed based on snapshots of associations between a few factors and health outcomes. In contrast, SDoH models and recent empirical studies suggest that multiple SDoH tend to co-occur and impact each other. For example, during the pandemic, Hispanic and Black or African American individuals not only had a higher exposure to COVID-19 due to their frontline jobs and overcrowded living conditions but also had a higher risk of serious infections due to previous health conditions not addressed due to lack of health care access [4]. Similarly, undocumented immigrants with lower incomes living in neighborhoods with high pollution, combined with the stress of deportation, have an increased risk of multiple chronic conditions such as depression and lung cancer [7]. Such studies have resulted in the Centers for Medicare and Medicaid Services emphasizing that SDoH are a multilevel construct that includes both individual and contextual factors that have complex interactions [23]. Furthermore, a distinction has been made between the aforementioned SDoH at the community level and social needs at the individual level [24]. However, while this distinction is critical when designing care pathways, there is growing consensus [25] that SDoH is an umbrella term that covers both levels, and it is an approach that has been adopted by both the PhenX Toolkit [21] and the *All of Us* surveys on SDoH widely used for analysis [9-11]. Therefore, we use *SDoH* to include all levels of nonmedical factors that impact health and well-being.

The aforementioned co-occurrences of multiple SDoH and their impact on health directly reflect the interconnected layers of the Dahlgren-Whitehead model shown in Figure 1. However, analysis of such co-occurrences and their health outcomes requires large datasets with multiple datatypes that have only recently been made available through the *All of Us* program.

### All of Us: Multiple Datatypes Across a Large Cohort of Underrepresented Americans

The *All of Us* research program [9-11] (*All of Us*), funded by the National Institutes of Health since 2015, aims to accelerate biomedical research to enable discoveries leading to individualized and equitable prevention and treatment. Such research is currently hampered due to the *limited range* of personal, clinical, social, and environmental variables available for the same individuals; *limited representation* in research datasets of socially marginalized populations; and *limited access* to individual-level data due to privacy laws.

To overcome these hurdles, *All of Us* provides three critical features: (1) a data repository that is projected to contain 1 million or more participants with data from multiple sources, including EHRs, health surveys, whole-sequence genomic data, physical measurements, and personal digital information such as from Fitbit trackers; (2) a cohort targeted to include 75% of participants from populations underrepresented in research (race, ethnicity, gender, sex, sexual orientation, and disability) oversampled from the US population; and (3) strictly enforced rules to prevent reidentification of participants by disallowing the download of any participant data or reporting of research results for subgroups of <20. These rules allow for analysis of the *All of Us* data to be categorized as non–human subjects research, which, combined with training and personal authentication by researchers, has resulted in a substantial reduction in administrative hurdles.

As of December 30, 2022 (Controlled Tier; version 6), *All of Us* contained 372,397 total participants, with 8.6% who had attempted all 9 health surveys (7 related to demographics and general health and 2 related to COVID-19) and 26.5% who had genomic data. Critical to this study is the recent addition of a survey specifically targeted to SDoH questions, which has been attempted by 15.5% in the *All of Us* cohort. A preliminary analysis revealed that SDoH appear to be distributed across multiple health surveys and EHR codes, with participants providing those data at different times on a rolling basis. However, little is known about the range of and responses to SDoH questions in *All of Us* and how SDoH co-occur to form subtypes, a critical step for selecting the methods to identify and interpret SDoH subtypes.

### Computational Methods to Identify and Interpret Subtypes

A wide range of studies [26-34] on topics ranging from molecular to environmental determinants of health have shown that most humans tend to share a subset of characteristics (eg, comorbidities, symptoms, and genetic variants), forming distinct subtypes (also referred to as *subgroups* or *subphenotypes* depending on the condition and variables analyzed). A primary goal of precision medicine is to identify such subtypes and infer their underlying disease processes to design interventions targeted to those processes [27,35]. Methods to identify subtypes include (1) investigator-selected variables such as race for developing hierarchical regression models [36] or assigning patients to different arms of a clinical trial, (2) existing classification systems such as the Medicare Severity Diagnosis Related Group [37] to assign patients to a disease category for purposes of billing, and (3) computational methods such as classification [38-40] and clustering [30,41] to discover subtypes.

Several studies have used computational methods to identify subtypes, each with critical trade-offs. Some studies have used *combinatorial* approaches [42] (eg, identify all pairs, all triples, and so on), which are intuitive but which can lead to a combinatorial explosion (eg, enumerating combinations of the 31 Elixhauser comorbidities would lead to 2^31^ or 2,147,483,648 combinations), with most combinations not incorporating the full range of symptoms (eg, the most frequent pair of symptoms ignores what other symptoms exist in the profile of patients with that pair). Other studies have used *unipartite* clustering methods [40,41] (clustering patients or comorbidities but not both together), such as k-means and hierarchical clustering, and dimensionality reduction methods, such as principal component analysis, to help identify clusters of frequently co-occurring comorbidities [42-48]. However, such methods have well-known limitations, including the requirement of inputting user-selected parameters (eg, similarity measures and the number of expected clusters) in addition to the lack of a quantitative measure to describe the quality of the clustering (critical for measuring the statistical significance of the clustering). Furthermore, because these methods are unipartite, there is no agreed-upon method to identify the patient subgroup defined by a cluster of variables, and vice versa.

More recently, bipartite network analysis [49] (see Multimedia Appendix 1 [48,50-55] for additional details) has been used to address the aforementioned limitations by automatically identifying *biclusters* consisting of patients and characteristics simultaneously. This method takes as input any dataset, such as *All of Us* participants and their SDoH, and outputs a quantitative and visual description of biclusters (containing both participant subgroups and their frequently co-occurring SDoH). The quantitative output generates the number and members of the biclusters, in addition to the statistical significance of the biclusteredness [50-52], and the visual output displays the quantitative information of the biclusters through a network visualization [53-55]. Therefore, bipartite network analysis enables (1) the automatic identification of biclusters and the significance of their biclusteredness and (2) the visualization of the biclusters critical for their clinical interpretability. Furthermore, the attributes of participants in a subgroup can be used to measure the subgroup risk of an adverse health outcome, develop classifiers for categorizing a new participant into one or more of the subgroups, and develop a predictive model that uses that subgroup membership for measuring the risk of an adverse health outcome for the classified participant.

However, while several studies [52,56-63] have demonstrated the usefulness of bipartite networks for the identification and clinical interpretation of subgroups, there has been no systematic attempt to identify SDoH subtypes, mainly because of the lack of large cohorts containing a wide coverage of SDoH. The *All of Us* program provides an opportunity to use bipartite networks for the identification and interpretation of SDoH subtypes using a wide range of variables in a large cohort and for analyzing their risk of adverse health outcomes, a critical step in advancing precision medicine.

## Methods

### Research Questions

Our analysis was guided by two research questions targeting the *All of Us* dataset:

1. What are the range of and responses to survey questions related to SDoH?
2. How do SDoH co-occur to form subtypes, and what are their risks for adverse health outcomes?

### Expert Panel

The selection of the research questions, variables, cohort, methods, and results and their interpretation were guided by an expert panel consisting of SDoH researchers with a professional background in applied demography, gerontology, and rehabilitation who worked closely with the machine learning and biostatistics researchers. The overall project and manuscript were examined by an ethicist for bias, stigma, and perpetuation of stereotypes. Therefore, the examination of each step in the project was aligned with the human-centered artificial intelligence approach [64-66].

### Data Description

#### Study Population

For question 1, we analyzed the full *All of Us* cohort (N=372,397) and characterized their responses to all the SDoH questions identified by the expert panel (described in the *Variables* section). For question 2, we analyzed all participants (n=12,913) who had valid responses to the SDoH questions identified in question 1 and used them to identify subtypes and their risks of specific outcomes.

#### Variables

For question 1, the expert panel was asked to review all 1113 questions across 6 *All of Us* non–COVID-19 health surveys, each of which was attempted once per participant (*The Basics*, *Lifestyle*, *Overall Health*, *Personal/Family Health History*, *Health Care Access and Utilization*, and *SDoH*), and the 2843 Systematized Medical Nomenclature of Medicine (SNOMED) codes related to SDoH [67]. The expert panel arrived at a consensus for the SDoH across the surveys and the SNOMED codes. As the SDoH-related SNOMED codes in the EHRs had very low use (see Multimedia Appendix 2 for a characterization), they were not further analyzed.

For question 2, to identify and analyze the SDoH subtypes, we used the following variables:

1. Independent variables included the SDoH factors identified from question 1.
2. Covariates included 3-digit zip code (to determine whether participants in each subtype came from a state that accepted Medicaid expansion, providing greater access to health insurance) and demographics (eg, age, sex, and race).
3. A total of 3 outcomes were included: *depression*, *delayed medical care*, and *emergency room (ER) visits in the last year*. *Depression* was selected as it is a common health outcome when individuals encounter SDoH in their daily lives, such as long-term stress resulting from racism [68], and the dysregulation of the hypothalamic-pituitary-adrenal axis [69]. Depression was defined as having a positive response to both of the following questions in *The Basics* survey—“Are you still seeing a doctor or health care provider for depression?” and “Has a doctor or health care provider ever told you that you have Depression?”—or having SNOMED codes related to depression in their EHRs (35489007, 36923009, 370143000, 191616006, or 66344007). *Delayed medical care* was selected as it often results from the lack of medical insurance, which can impact the use of medical care when needed, leading to poorer health outcomes [70]. Delayed medical care was defined as having one or more positive responses to 9 survey questions (delayed care due to transportation, rurality, nervousness, work, childcare, copay, older adult care, out-of-pocket costs, and deductible costs) in the *Health Care Access* and *Utilization* survey. *Emergency room (ER) visits in the last year* was selected because lack of medical insurance often results in individuals not seeking early medical care when needed, leading to an exacerbation of conditions precipitating one or more ER visits [71]. As the survey questions that we used for SDoH subtyping were based on outcomes in the previous year, we defined ER visits for a participant as having one or more ER visits (current procedural terminology 99281-99285) 1 year preceding the date when the SDoH survey was completed.

### Analytical Approach

#### Question 1: What Are the Range of and Responses to Survey Questions Related to SDoH?

#### Identification and Coding of SDoH

**Figure 2:**
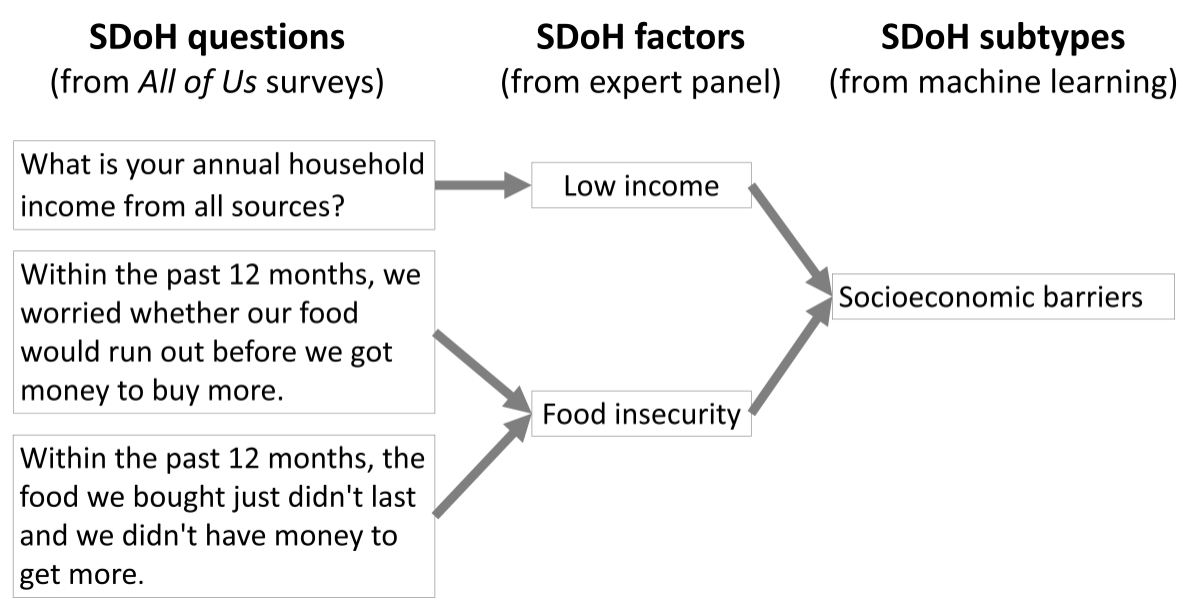
Examples showing how the social determinants of health (SDoH) questions from the *All of Us* surveys that differed in their levels of granularity were transformed by the expert panel into SDoH factors with uniform granularity to ensure consistency for analysis and interpretation and clustered into SDoH subtypes through machine learning. The SDoH questions and factors were subsequently analyzed for coverage across the 5 Healthy People 2030 domains.

To analyze the range and responses to the survey questions, we first characterized all SDoH in *All of Us* at two levels of granularity—(1) SDoH questions based on the surveys used to collect the data and (2) SDoH factors, which were categories of the SDoH questions to form a coarser-grained classification (see Figure 2, which explains SDoH questions, factors, and subtypes).

To identify the SDoH questions, members of the expert panel independently used their domain knowledge about SDoH to identify and code the SDoH questions and examine their range with respect to the 5 HP-30 domains using the following steps: (1) reviewed all 1113 questions across 6 health surveys (excluding 2 related to COVID-19) and extracting all SDoH questions that were relevant, (2) transformed all positive or value-free questions into negative phrases and abbreviating them for interpretability in the graphs (eg, *How often do you have someone help you read health-related materials?* was changed to *No one to help read health materials*), (3) reverse coded and dichotomized the abbreviated SDoH questions (eg, always or often=1 and never, occasionally, or sometimes=0), and (4) categorized the SDoH questions into 1 of the 5 HP-30 SDoH domains (economic stability, education access and quality, health care access and quality, neighborhood and built environment, and social and community context). The expert panel subsequently met and collaboratively resolved any differences among their coding schemes to arrive at a consensus (see Multimedia Appendix 3 for the 110 SDoH questions and their consensus coding by the expert panel).

To characterize the SDoH factors, the expert panel arrived at a consensus to categorize one or more of the aforementioned SDoH questions in *All of Us* into SDoH factors and examined their range with respect to HP-30 using the following steps: (1) reviewed the subgrouping labels of questions in the *All of Us* surveys and integrating them to categorize the SDoH into factors, (2) coded a participant as having a “1” for an SDoH factor if they answered one or more of the questions within that factor with a “1,” and (3) categorized the SDoH factors into 1 of the 5 HP-30 SDoH domains (economic stability, education access and quality, health care access and quality, neighborhood and built environment, and social and community context; see Multimedia Appendix 3 for the 110 SDoH questions, their consensus coding into 19 SDoH factors, and mapping to the 5 SDoH domains from HP-30).

#### Analysis of the Range and Responses to SDoH Questions and Factors

The aforementioned knowledge-based classification of SDoH questions and SDoH factors was analyzed to examine their range (with respect to the 5 HP-30 domains) and their responses (across all participants in *All of Us*) using the following four methods: (1) bar graph displaying the number of participants who had valid answers (all responses other than “skip” or “choose not to answer”) to each of the SDoH questions, sorted by survey based on mean response, and then sorted by raw response within each survey. Finally, to analyze their range, each bar was colored to denote one of the 5 SDoH domains defined by HP-30; (2) Venn diagram showing how many participants had cross-sectionally valid responses to all identified SDoH questions or factors; (3) table describing the number and proportion of race, ethnicity, sex, gender, and age between those who answered the SDoH questions or factors and those who did not have valid responses; and (4) frequency distribution of the number of SDoH questions or factors across participants who had valid responses for all the SDoH questions. The aforementioned plots are shown in the *Results* section.

#### Question 2: How Do SDoH Co-Occur to Form Subtypes, and What Are their Risks for Adverse Health Outcomes?

##### Data

We used the cohort identified in question 1 (participants who had valid answers to all the SDoH questions). However, examination of the SDoH questions revealed that some of them (eg, *cannot afford dental care* and *cannot afford prescriptions*) had a finer level of granularity compared to others (eg, single household). As the questions with a finer level of granularity tend to be more strongly corelated to each other in comparison to other coarser-grained questions, they also tend to cluster together more strongly, confounding the interpretation of the subtypes. In contrast, as the SDoH factors had a more uniform granularity and were at a level of abstraction that was appropriate to guide referral to the proper social services, we used them to identify the SDoH subtypes.

##### Analytical Model

###### Overview

**Figure 3:**
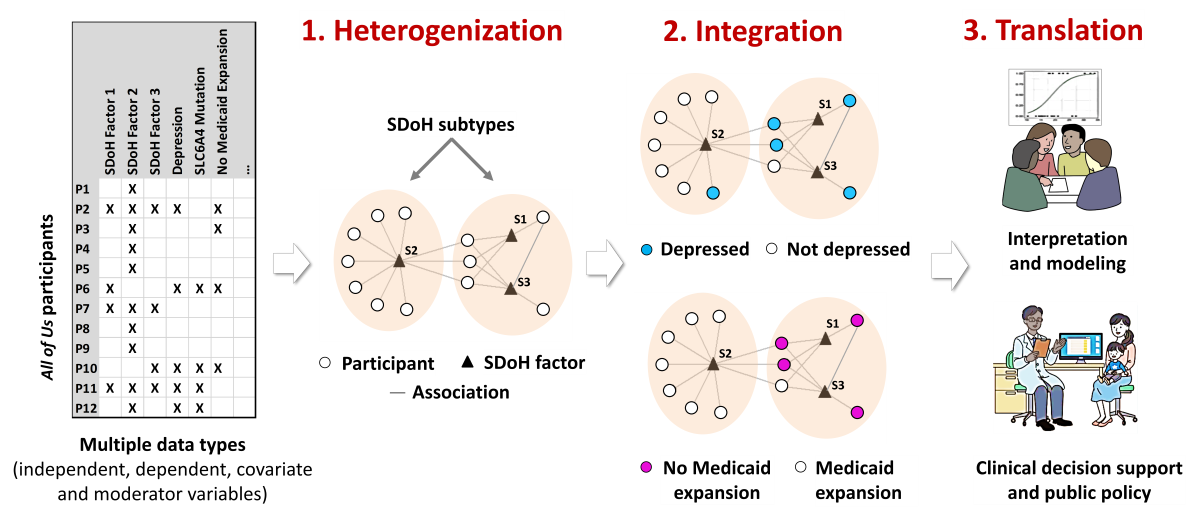
The three steps of the heterogenization, integration, and translation framework to analyze social determinants of health (SDoH): (1) heterogenization of the data to identify subtypes, (2) integration of multiple datatypes such as from electronic health records (eg, depression) and information on state of residency (eg, to determine Medicaid expansion) to determine the risk and enrichment of each subtype, and (3) translation of subtypes through interpretation and predictive modeling with the goal of designing clinical decision support systems and public policy. SLC6A4: Serotonin Transporter Gene Polymorphism.

To identify SDoH subtypes, their associations with outcomes and covariates, and their future translation into precision medicine and public policies, we used a 3-part analytical framework called heterogenization, integration, and translation (HIT). As shown in Figure 3, the *heterogenization* step was used to identify the subtypes through the use of bipartite modularity maximization [50-52] (see Multimedia Appendix 1 for more details), the *integration* step was used to measure the association of each subtype with multiple datatypes [72], and the *translation* step was used to qualitatively interpret the subtypes [72] with the goal of developing in the future a decision support system to translate the subtypes into clinical practice and the design of public policies. The following sections describe the specific methods used in each of the HIT steps.

###### Heterogenization: Identification of Subtypes

As there were many participants who did not have valid answers to the SDoH questions, dropping them resulted in differences in the proportion of demographic variables compared with the full *All of Us* cohort. Therefore, the data needed to be adjusted to better reflect the overall *All of Us* participants. To adjust the demographic distribution of the cohort to match the full *All of Us* cohort, we used inverse probability weighting (IPW) [73,74]. IPW calculates weights to proportionally boost the values of participants who are underrepresented in a cohort with respect to a comparison, such as the full *All of Us* dataset, using a method similar to that of an earlier study on *All of Us* [75] (Multimedia Appendix 4). Next, we multiplied the IPW-generated weights with the original binary values for each participant in our cohort and used the min-max method to range-normalize those weights within each SDoH factor. Finally, to test the replicability of the SDoH factor biclustering, we randomly divided the dataset into a training and a replication dataset.

We identified subtypes in the training dataset and tested the degree to which the SDoH factor co-occurrences were replicated in the replication dataset using the following steps: (1) modeled participants and SDoH factors as a weighted bipartite network (step 1 in Figure 3) where nodes were either participants (circles) or SDoH factors (triangles) and the associations between participant–SDoH factor pairs were weighted edges (lines) generated from IPW (the inclusion of IPW-generated weights enabled the network to represent the demographic distribution of the full *All of Us* dataset), (2) used a bipartite modularity maximization algorithm [50-52] (which takes edge weights into consideration) to identify the number of biclusters and their members and measure the degree of biclusteredness through bicluster modularity (*Q*, defined as the fraction of edges falling within a cluster minus the expected fraction of such edges in a network of the same size with randomly assigned edges), (3) measured the significance of *Q* by comparing it to a distribution of the same quantity generated from 1000 random permutations of the network while preserving the network size (number of nodes) and the distribution of weighted edges for each participant, (4) used the Rand index (RI) to measure the degree to which SDoH occurred and did not co-occur in the same cluster in the training and replication datasets, and (5) measured the significance of the RI by comparing it to the mean of a distribution of the same quantity generated by randomly permuting the training and replication datasets 1000 times while preserving the size of the networks and the distribution of weighted edges for each participant.

###### Integration: Risk and Enrichment of Subtypes

We used logistic regression to measure the odds ratio (OR) for each subtype compared pairwise to each of the other subtypes for the 3 outcomes (depression, delayed medical care, and ER visits in the last year) and for living in a state with no Medicaid expansion. To adjust for the difference in demographics due to the missingness, we used weights generated from IPW for each participant, and the comparisons were adjusted for demographics (eg, age, sex, and race) and corrected for multiple testing within each outcome using false discovery rate. As 13.07% (1688/12,913) of the participants did not have 3-digit zip code information, we used IPW to measure the weights of the cohort and used them to account for potential sample selection bias.

###### Translation: Interpretation of Subtypes

The subtype interpretation was done using the following steps: (1) used the *Fruchterman-Reingold* [53] and *ExplodeLayout* [54,55] algorithms to visualize the bipartite network along with the risk of each of the outcomes; (2) asked the expert panel to independently label the subtypes, infer the mechanisms that increase the risks in each subtype for the 3 outcomes (depression, delayed medical care, and ER visits in the last year) with potential strategies to reduce those risks, and then collaboratively come to a consensus; and (3) asked an ethicist to examine the results and their interpretations for bias, stigma, and perpetuation of stereotypes.

#### Ethical Considerations

The original data collection by the *All of Us* program was approved by an institutional review board as described on the web [76].

The secondary analysis of the *All of Us* data conducted in this work did not receive approval or exemption from an institutional review board. Such an approval or exemption is not required as described on the web [77].

Therefore, the authors had permission to conduct a secondary analysis of the data.

## Results

### Question 1: What Are the Range of and Responses to Survey Questions Related to SDoH?

#### Identification and Coding of SDoH Questions and Factors

The expert panel identified 110 questions from 4 surveys (*The Basics*, *Overall Health*, *Healthcare Access and Utilization*, and *SDoH*). Of these 110 questions, 110 (100%) were abbreviated, and 48 (43.6%) were negatively worded and coded (Multimedia Appendix 3). The 110 SDoH questions were further categorized into 19 SDoH factors (one of these was *Delayed medical care*, which was used as an outcome).

#### Responses to SDoH Questions and Factors

**Figure 4:**
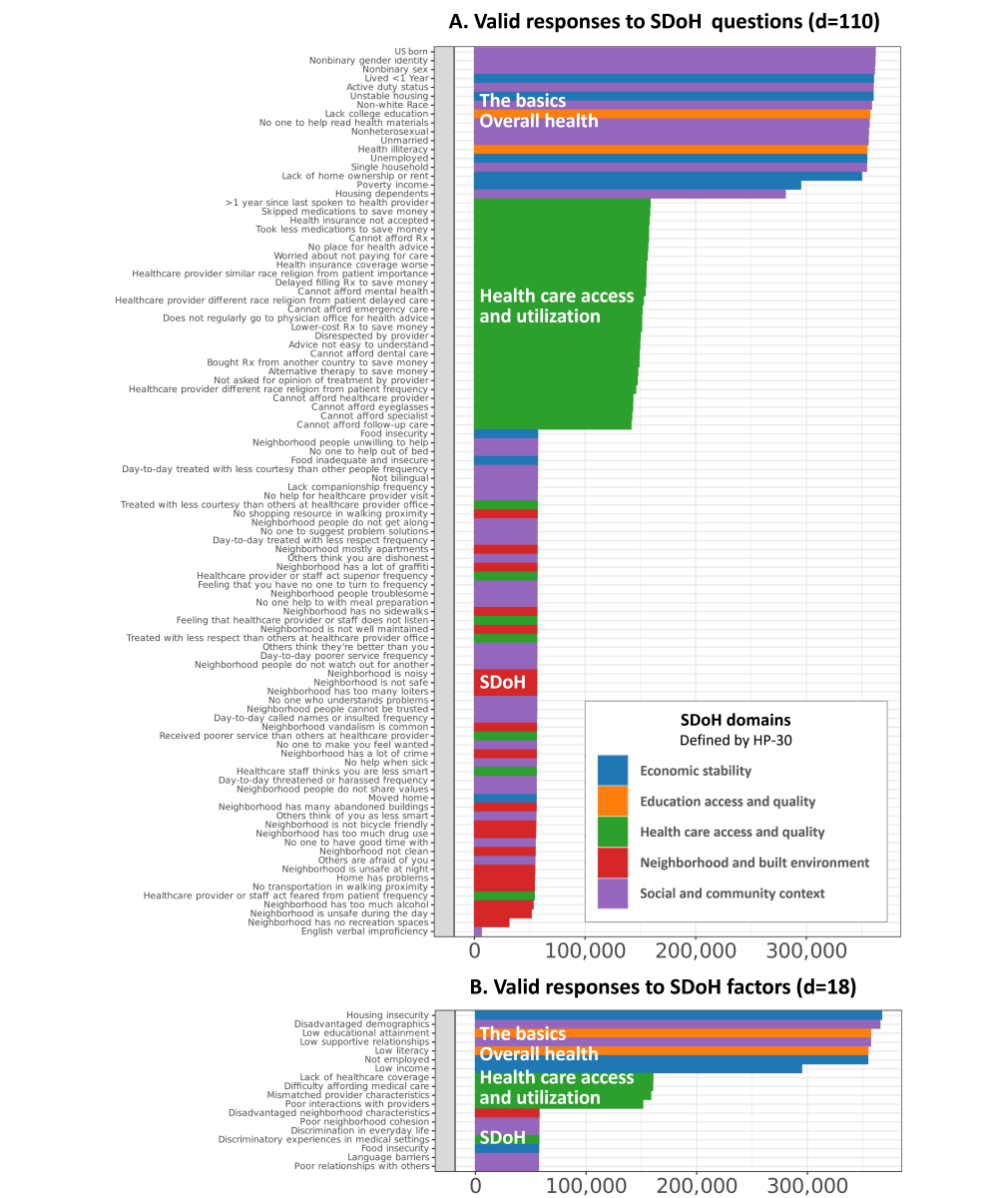
The number of valid responses for (A) 110 social determinants of health (SDoH) questions and (B) 18 SDoH factors. The colors denote how the SDoH in each were categorized based on the 5 Healthy People 2030 (HP-30) domains. Rx: prescription medication.

As shown in Figure 4A, the number of valid responses to each of the 110 SDoH questions was largely dictated by the surveys in which the responses were solicited. SDoH from 2 surveys (*The Basics* and *Overall Health*) had the most valid responses (mean 349,434, SD 23,556), followed by *Healthcare Access and Utilization* (mean 149,898, SD 6146) and, finally, the *SDoH* survey (mean 55,960, SD 1083). This pattern of responses matched how answers to each of the surveys were solicited—at enrollment, all participants are required to do *The Basics* and *Overall Health* surveys, and then, on a rolling basis, responses to the other surveys are solicited. The *SDoH* survey was the last survey that was solicited, which explained it having the lowest number of responses. As shown in Figure 4B, this pattern of missingness held for the responses at the SDoH factor level, which was not unexpected as the SDoH factors were aggregations of the SDoH questions. However, as shown in Figures 4A and 4B by the uneven number of valid responses within each survey block, there were several SDoH questions that had invalid responses (“skip” or “choose not to answer”) at both levels of granularity: *The Basics* (339,254/5,655,412, 5.99%), *Healthcare Access and Utilization* (341,516/5,587,957, 6.11%), *Overall Health* (32,669/744,126, 4.39%), and *SDoH* (83,699/3,206,035, 2.61%). Furthermore, the proportion of valid to invalid responses among them was significantly different for the SDoH questions (N=365,237, χ^2^_2_=57.5; *P*<.001) and for the SDoH factors (N=372,063, χ^2^_2_=75.6; *P*<.001).

#### Range of SDoH Questions and Factors

As shown by the colored bars in Figure 4, the surveys spanned the full range of the 5 SDoH HP-30 domains. The SDoH questions in *The Basics* and *Overall Health* surveys were predominantly related to economic stability (blue) and social and community context (purple), those in the *Healthcare Access and Utilization* survey were all related to that topic (green), whereas those from the *SDoH* survey were a mix of all 4 domains. Overall, the 4 surveys contained 110 SDoH questions that together had 100% coverage of the 5 HP-30 domains (social and community context: n=38, 34.5%; neighborhood and built environment: n=19, 17.3%; economic stability: n=10, 9.1%; education access and quality: n=2, 1.8%; health care access and quality: n=42, 38.2%). This characterization suggests that, while the SDoH in *All of Us* have broad domain coverage across the surveys, analysis of them requires access to all 4 surveys, each of which has different levels of completion and valid responses.

#### Cohort With Maximized Valid Responses

**Figure 5:**
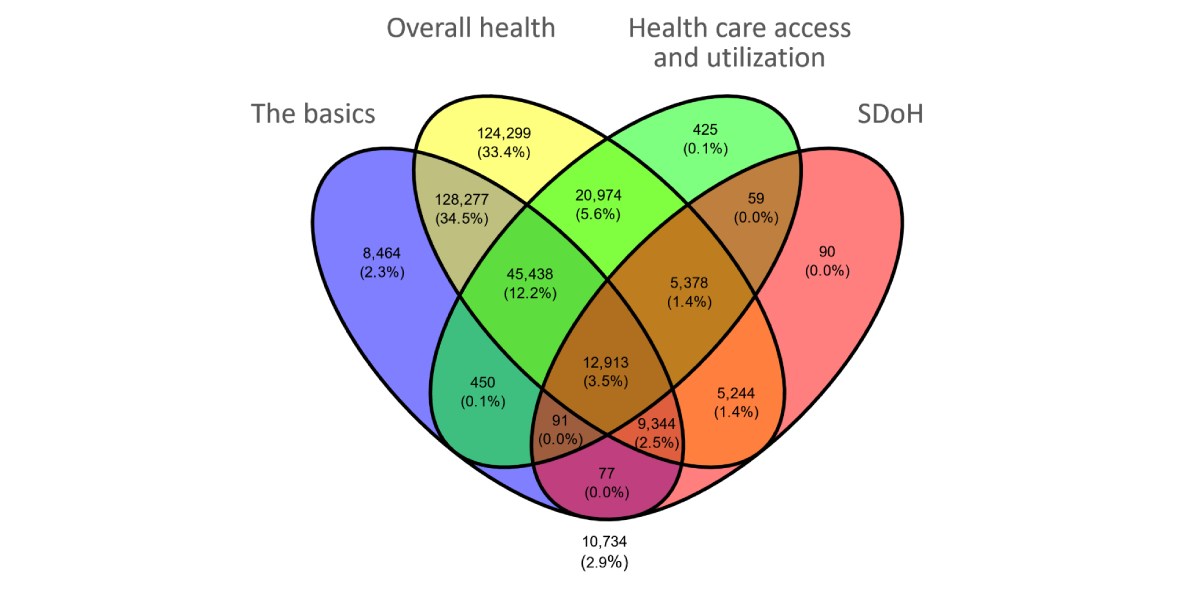
Venn diagram showing 3.47% (12,913/372,397) of participants of the full cohort who had valid responses to all 98 social determinants of health (SDoH) questions.

Given the large degree and systematic nature of missingness in 2 of the 4 surveys, we could not use multiple imputation to estimate the values. Therefore, we had to find a subset of participants who had valid responses to all the SDoH questions. An examination revealed that 2 SDoH questions had <10% of responses (*English verbal frequency*: 6193/371,942, 1.67%; *Neighborhood has no recreation spaces*: 31,152/371,942, 8.38%), accounting for the largest loss in cohort size with valid responses. Therefore, these questions were dropped from further analysis. Furthermore, one question required a branched response (*Living situation* branching to *Did not live in a house*), and these responses were merged. Finally, as we used *Delayed medical care* as an outcome, 8.2% (9/110) of the questions related to that topic were removed, resulting in a total of 98 SDoH questions. As shown in Figure 5, a Venn diagram of the overlap among the valid responses across the surveys revealed that 3.47% (12,913/372,397) of the participants had valid responses to all 98 SDoH questions.

#### Co-Occurrence of the Number of SDoH Across Responders

**Figure 6:**
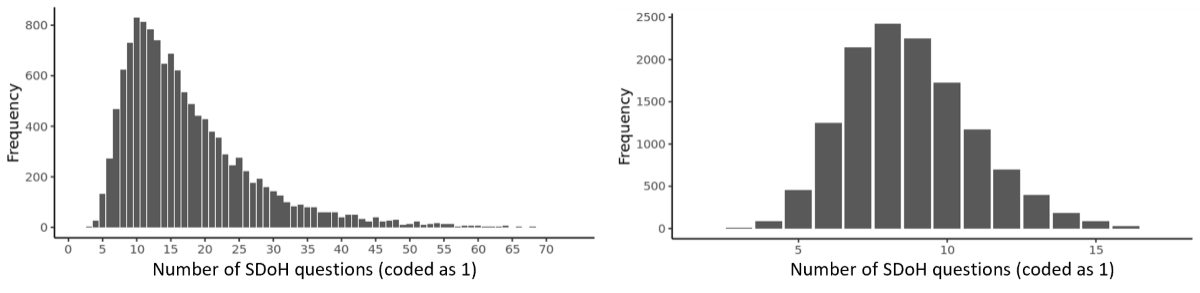
Frequency distribution of (A) number of co-occurring responses to social determinant of health (SDoH) questions across the 12,913 participants with valid answers to the 98 SDoH questions and (B) number of co-occurring SDoH factors across 19 SDoH factors.

As shown in Figure 6, participants had a median of 15 SDoH question co-occurrences and a median of 9 SDoH factor co-occurrences. Furthermore, participants of racial and ethnic minority groups who had valid responses to the 110 SDoH questions had a significantly higher median number of co-occurring SDoH compared to the equivalent White population (median 20 for participants of racial and ethnic minority groups; median 14 for White participants; *P*<.001). These results show the high co-occurrences of SDoH at both levels of granularity, with a significant difference in median co-occurrences between the White participants and the participants of racial and ethnic minority populations with valid responses.

#### Participant Demographics With Valid Responses to SDoH Questions

As the cohort size dropped to 3.47% (12,913/372,397), we analyzed how that impacted the demographic distribution compared with the overall *All of Us* dataset. As shown in Table 1, there were statistically significant differences in race (N=372,397, χ^2^_5_=2073.1; *P*<.001) and ethnicity (N=372,397, χ^2^_9_=6292.2; *P*<.001) between the 2 cohorts after multiple testing correction, with a higher proportion of White participants having valid answers than participants of racial or ethnic minority groups. Furthermore, there was a statistically significant difference in age between the participants who had valid answers and those who did not (*H*1=148.08; *P*<.001). These results show the demographic differences between the cohort with complete and valid answers to the SDoH questions and the full *All of Us* dataset, necessitating the use of IPW–generated weights to address those imbalances, as discussed in question 2.

**Table 1:** The demographic differences between the total *All of Us* (AoU) participants and those who had valid answers to all 110 social determinant of health (SDoH) questions (N=372,397). Participant counts of <20 are shown as a count of 20 based on the AoU reporting rules.

Demographics		Total AoU participants	Total AoU participants with valid^a^ SDoH answers (n=12,913)
**Race, n (%)**			
	Asian	12,459 (3.35)	324 (2.51)
	Black or African American	73,383 (19.71)	482 (3.73)
	White	201,149 (54.01)	11,279 (87.35)
	Other or >1 population	26,890 (7.22)	343 (2.66)
	None indicated	58,516 (15.71)	485 (3.76)
**Ethnicity, n (%)**			
	Not Hispanic or Latino	288,227 (77.4)	12,095 (93.67)
	Hispanic or Latino	66,704 (17.91)	751 (5.82)
	Additional options	17,466 (4.69)	67 (0.52)
**Sex at birth, n (%)**			
	Female	222,495 (59.75)	8236 (63.78)
	Male	138,831 (37.28)	4674 (36.2)
	Intersex	80 (0.02)	20 (0.15)
	Additional options	10,991 (2.95)	20 (0.15)
**Gender, n (%)**			
	Female	220,833 (59.3)	8113 (62.83)
	Male	138,140 (37.09)	4642 (35.95)
	Nonbinary	920 (0.25)	60 (0.46)
	Transgender	464 (0.12)	20 (0.15)
	Additional options	12,040 (3.23)	79 (0.61)
**Age (y), median (range)**		56 (19-122^b^)	58 (19-93)

^a^Participants who completed all questions and did not skip or choose not to answer a question.

^b^Age of 122 years=a participant chose the earliest birth year (1900).

### Question 2: How Do SDoH Factors Co-Occur to Form Subtypes, and What Are Their Risks for Adverse Health Outcomes?

#### Overview

The cohort used to identify the subtypes consisted of 12,913 participants, of whom 12,886 (99.79%) had valid IPW–generated weights. The latter cohort was split randomly into the training and replication datasets, each with complete data for 18 SDoH factors (identified in question 1) in addition to the 3 outcomes (depression, delayed medical care, and ER visits in the last year) and covariates (demographics). The results are organized based on the 3 parts of the HIT framework described in Figure 3.

#### Heterogenization: Identification of Subtypes

**Figure 7:**
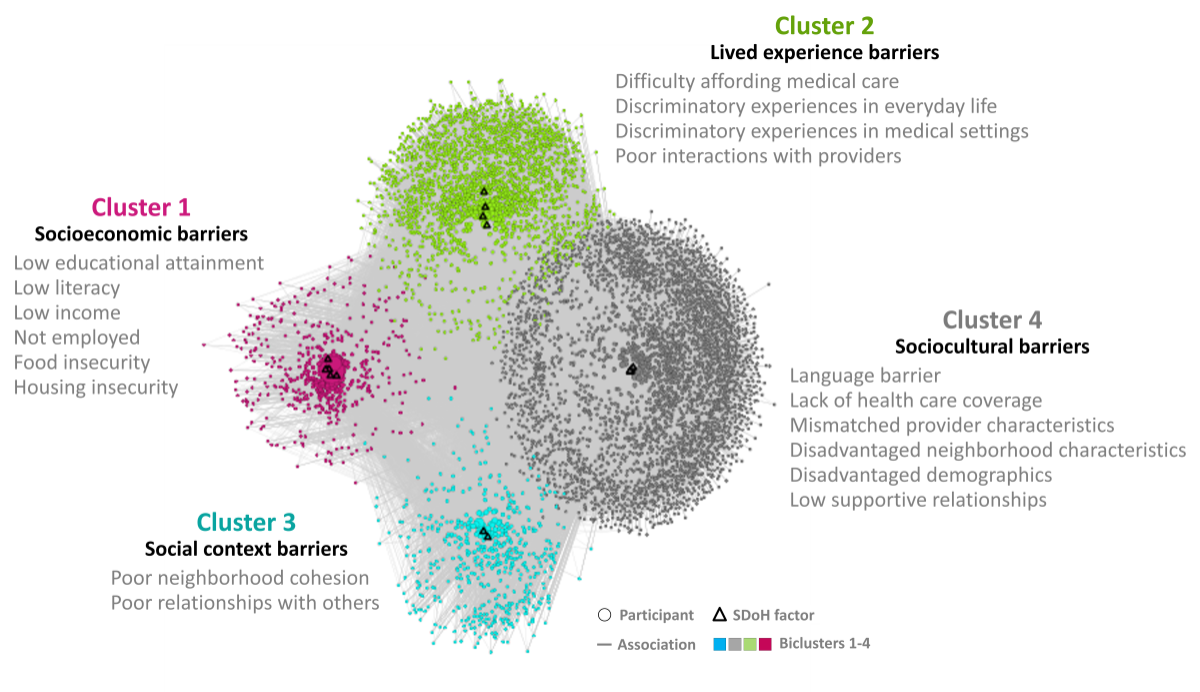
A total of 4 biclusters in the training dataset consisting of subgroups of participants (n=6492) and their most frequently co-occurring social determinant of health (SDoH) factors (d=18).

**Figure 8:**
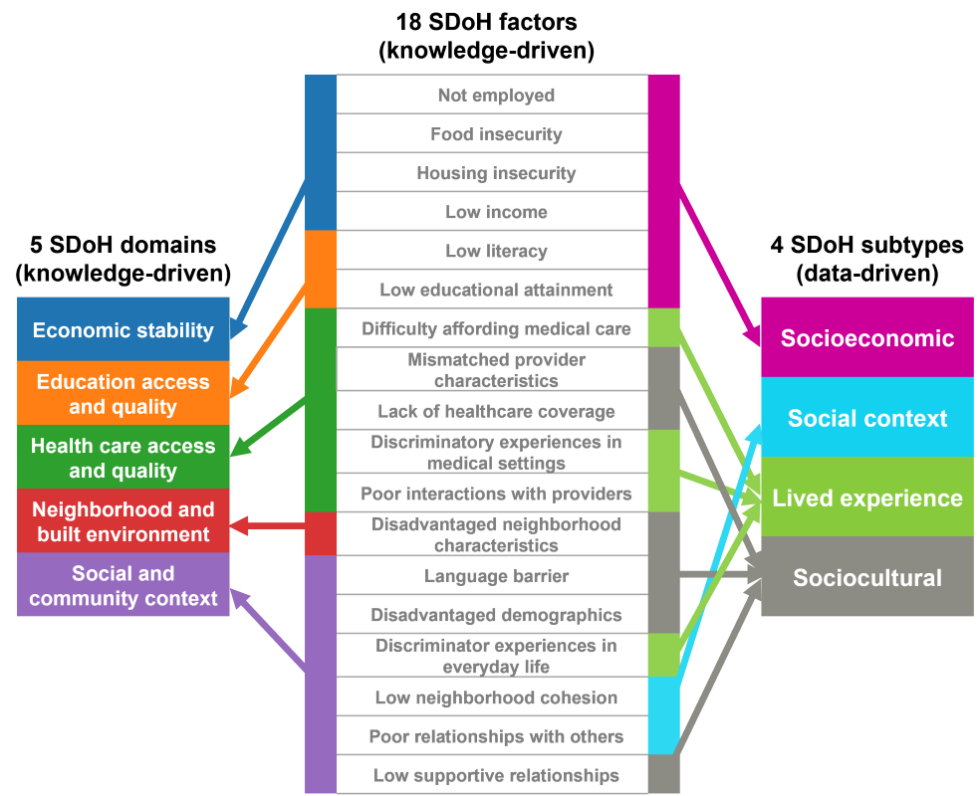
A total of 18 social determinant of health (SDoH) factors (center) have a hierarchical relationship with the 5 SDoH domains defined by Healthy People 2030 (HP-30; left), both of which are knowledge driven. In contrast, the data-driven analysis shows that SDoH factors have a complex relationship with the SDoH subtypes (right) identified through machine learning (ML), reflecting how they co-occur in the real world and aligned with models such as the Dahlgren-Whitehead model.

The subtypes were identified by using a bipartite network where the edges were weighted using the IPW-generated weights to account for the imbalance in demographics between our cohort and the full *All of Us* dataset. The weighted bipartite network of the training dataset (n=6492) and the 18 SDoH factors revealed 4 biclusters with statistically significant bicluster modularity (*Q*=0.13; random-*Q*=0.11; *z*=7.5; *P*<.001). As shown in Figure 7, there were 4 clusters with participant subgroups and their most frequently co-occurring SDoH factors (*Cluster 1 [pink]*: low educational attainment, low literacy, low income, not employed, food insecurity, and housing insecurity; *Cluster 2 [green]*: difficulty affording medical care, discriminatory experiences in everyday life, discriminatory experiences in medical settings, and poor interactions with providers; *Cluster 3 [blue]*: poor neighborhood cohesion and poor relationships with others; and *Cluster 4 [gray]*: language barrier, lack of health care coverage, mismatched provider characteristics, disadvantaged neighborhood characteristics, disadvantaged demographics, and low supportive relationships). These co-occurrences of SDoH factors were significantly replicated in the replication dataset (real RI=0.88; random RI=0.62; *P*<.001). As shown in Figure 8, while the 18 SDoH factors have a hierarchical relationship with the 5 *knowledge-driven* HP-30 domains (shown on the left), those same SDoH factors have a more complex relationship with the 4 *data-driven* biclusters (shown on the right).

#### Integration: Risk and Enrichment of Subtypes

Table 2 shows the association of each subtype with the 3 outcomes. As shown by the italicized row, Cluster 1 (low educational attainment, low literacy, low income, not employed, food insecurity, and housing insecurity) had a significantly higher OR for each of the 3 outcomes than Cluster 4 (mismatched provider characteristics, disadvantaged neighborhood characteristics, lack of health care coverage, disadvantaged demographics, low supportive relationships, and language barriers). Furthermore, within the Depression outcome, each of the clusters had a significantly higher OR than one other cluster, forming a ranking of risk among all the 4 clusters (1>3>2>4). In contrast, Delayed medical care had 2 other significant associations (2>1 and 3>4), with ER visits in the last year having only 1 significant pairwise association that fit into the overall trend.

As shown in Table 3, this trend continued in the enrichment analysis of association with living in a state with *No Medicaid expansion*. As shown, Cluster 1 had a significantly higher OR than Cluster 4 in addition to having a significantly OR than the other clusters. The overall results suggest that Cluster 1 and Cluster 4 form “book ends” representing the high and low ends of the risk spectrum among the clusters.

**Table 2:** Cluster comparisons to measure the risk across all 3 outcomes. Cluster 1 had a significantly higher risk than Cluster 4 for all 3 outcomes (shown in italics). The depression outcome had a distinct ranking of risks, whereas the other 2 outcomes had a subset of that ranking. All *P* values shown are corrected for multiple testing.

Cluster comparison	Outcomes					
	Depression		Delayed medical care		ER^a^ visits in the last year	
	OR^b^ (95% CI)	*P* value	OR (95% CI)	*P* value	OR (95% CI)	*P* value
1 vs 2	1.7 (1.5-2)	<.001	0.78 (0.67-0.92)	.004^c^	1.2 (0.91-1.6)	.24
1 vs 3	1.3 (1.1-1.6)	.02^d^	0.88 (0.72-1.1)	.23	1.4 (0.96-1.9)	.13
*1* vs *4*	4.2 (3.5-5.1)	<.001	3.5 (3-4.1)	<.001	1.8 (1.4-2.3)	<.001
2 *vs* 3	0.79 (0.64-0.97)	.02^d^	1.2 (0.98-1.4)	.09	1 (0.75-1.5)	.80
2 vs 4	2.3 (1.9-2.7)	<.001	4.3 (3.7-5)	<.001	1.3 (1-1.7)	.12
3 vs 4	2.9 (2.3-3.5)	<.001	3.6 (3-4.4)	<.001	1.4 (0.95-1.9)	.13

^a^ER: emergency room.

^b^OR: odds ratio.

^c^*P*<.01.

^d^*P*<.05.

**Table 3:** Cluster 1 had a significantly higher odds ratio (OR) than Cluster 4 (shown in italics) for No Medicaid expansion in addition to having a higher OR than Cluster-2 and Cluster-3. All *P* values shown are corrected for multiple testing.

Cluster comparison	Enrichment—No Medicaid expansion	
	OR (95% CI)	*P* value
1 vs 2	1.5 (1.3-1.8)	<.001
1 vs 3	1.3 (1-1.6)	.048^a^
*1 vs 4*	*1.3 (1.1-1.5)*	*.006* ^b^
2 vs 3	0.99 (0.8-1.2)	.97
2 vs 4	0.99 (0.86-1.2)	.97
3 vs 4	1 (0.82-1.2)	.97

^a^*P*<.05.

^b^*P*<.01.

#### Translation: Interpretation of SDoH Subtypes and Design of Potential Interventions

The expert panel examined the co-occurrences of SDoH factors within each bicluster shown in the network visualization (Figure 7) and integrated them with the quantitative ORs in Tables 2 and 3. The consistent “book ends” result where Cluster 1 had significantly higher ORs than Cluster 4 across all 4 variables was of strong interest and interpreted as follows: (1) Cluster 1 was labeled *Socioeconomic barriers* as it comprised multiple high-risk SDoH. These co-occurring SDoH could have resulted from cascades over time, such as low educational attainment, potentially leading to lower rates of employment and lower income with higher rates of food and housing insecurity. Such cascading factors can be perceived as being relatively unmodifiable, leading to a higher risk of chronic stress and depression. Furthermore, the strong association of this subtype with the outcomes *Delayed medical care* and *ER visits in the last tear* and the fact that participants in this subtype were more likely to be from a US state with *No Medicaid expansion* provided a more comprehensive understanding of this high-risk SDoH subtype; (2) Cluster 4 was labeled *Sociocultural barriers* as it contained a combination of SDoH related to disadvantaged neighborhood characteristics and low supportive relations in addition to language barriers and mismatched provider interactions. In contrast to the socioeconomic barriers in Cluster 1, many of the sociocultural barriers could be perceived as potentially modifiable, resulting in a lower risk of depression, delayed medical care, and ER visits. Participants who matched this profile could be screened for language and communication barriers, useful for providing culturally competent care, identifying providers who better match the profile of the individuals, and providing resources to facilitate contact with matching nationality or cultural groups in the vicinity or on the web.

While Clusters 1 and 4 formed the “book ends” of risk across the 3 outcomes, potentially caused by relative differences in the unmodifiability of their frequently co-occurring SDoH, Cluster 2 was flagged as critical and labeled *Lived experience barriers*. The SDoH in this cluster included discriminatory experiences in everyday life and in medical settings in addition to poor interactions with providers and difficulty affording medical care. These frequently co-occurring SDoH could explain why this subtype had a significantly higher OR for *Delayed medical care* than Cluster 1. Finally, Cluster 3 was labeled *Social context barriers* as the SDoH were related to poor neighborhood cohesion and relationships with others. While not as critical as Clusters 1 and 2, this cluster still had a significantly higher OR for Depression than Cluster 4. Together, the 4 clusters could explain how different degrees of unmodifiability in frequently co-occurring SDoH might impact health outcomes.

The expert panel and the ethicist concluded that clinicians treating patients who match each subtype profile could be alerted of specific risks and, consequently, motivate a discussion about mental health and consequences of delayed medical care with the goal of collaboratively exploring options and solutions with the patients. The results could also be useful for resource planning in hospitals to ensure that there is adequate staff to address the needs of the populations they serve, and for proposing health care policies to address the critical connection between specific combinations of SDoH and their impact on public health. For example, many health equity policies categorize Americans based on sociodemographic variables like race and income, which are proxies of need rather than the needs themselves. Instead, such policies could categorize Americans based on SDoH subtypes and their risks to more precisely allocate resources based on combinations of real-world needs.

Furthermore, the subtypes did not have a one-to-one mapping to the 5 SDoH domains defined by HP-30. As shown in Figure 8, these data-driven clusters have a complex relationship with the SDoH domains and factors. While one subtype belonged to a single domain (the *Social context* subtype belonged to the *Social and community context* domain), 3 of the 4 subtypes belonged to ≥2 domains (eg, the *Socioeconomic barriers* subtype belonged to the *Economic stability* and *Education access and quality* domains). Such interdomain relationships reflect how SDoH co-occur in the real world, reflecting the complex cross-domain interactions described in the Dahlgren-Whitehead model (Figure 1). These relationships could be useful for refining conceptual models to explain the complex association between SDoH and adverse health outcomes and build more accurate SDoH models for predicting adverse health outcomes.

## Discussion

### Principal Findings

The mechanisms through which SDoH precipitate adverse health outcomes are complex, consisting of many interacting factors and feedback loops among individual and environmental and contextual factors. While this phenomenon has been studied for >3 decades, critical hurdles for researchers have included the *limited range* of datatypes, *limited representation* of populations that have been socially marginalized, and *limited access* to individual-level data at scale due to privacy laws. Recognizing that *All of Us* has well-articulated plans and resources to overcome these limitations but is still in a rapidly evolving stage, we conducted a systematic characterization of >100 SDoH survey questions available in *All of Us* and used them to identify SDoH subtypes with the future goal of designing targeted interventions.

For question 1, we identified 110 SDoH questions across 4 surveys, which covered all 5 domains in HP-30. However, the results also revealed a large degree of missingness in survey responses (1.76%-84.56%), with later surveys having significantly fewer responses than earlier ones, and significant differences in race, ethnicity, and age of participants among those who completed the surveys with SDoH questions compared to those in the full *All of Us* dataset. Furthermore, as the SDoH questions varied in granularity, they were categorized by an expert panel into 18 SDoH factors. For question 2, the subtype analysis identified 4 biclusters with significant biclusteredness and significant replication. Furthermore, there were statistically significant associations between specific subtypes and the outcomes, as well as with Medicaid expansion, each with meaningful interpretations and potential targeted interventions. Finally, the identified subtypes spanned one or more HP-30 domains, revealing the difference between the current knowledge-based SDoH domains and the data-driven subtypes.

While the results revealed the nature of and responses to SDoH questions in *All of Us* and significant and interpretable SDoH subtypes, the analysis revealed critical opportunities and challenges related to data, methods, and theory. Such insights are useful for future researchers conducting similar analyses on *All of Us* and, therefore, are discussed in the following sections.

### Data: Missingness and Granularity

#### Missingness

The analysis revealed 3 types of missingness. The first was *rollout missingness*. This type of missingness was largely dictated by how the surveys were rolled out to participants. As all participants at enrollment are required to do *The Basics* and *Overall Health* surveys, these surveys had the highest number of responses, followed by the later solicited *Healthcare Access and Utilization* and *SDoH* surveys rolled out more recently in 2022. This order of rollout was the main source of missingness, resulting in a precipitous reduction in cohort size for those who had answers to all the SDoH questions. The second type was *valid answer missingness*. As participants can choose not to answer any survey questions, the data contained “skip” and “choose not to answer” responses. However, these responses accounted for a much smaller reduction in cohort size for complete data than rollout missingness. The third type was *low use missingness*. Although there were 259 SDoH SNOMED codes, only 93 (35.9%) had such information for >20 participants that are allowed to be reported. This could be because most clinicians currently do not screen for SDoH as it is typically done by a social worker. Furthermore, we also attempted to use 3-digit zip codes to determine which subtypes had a significant association with living in a state that did not offer Medicaid expansion. However, 13.07% (1688/12,913) of the participants did not have zip code information (which was adjusted by using IPW).

Together, the aforementioned 3 types of missingness impacted the size of the resulting cohort that had valid answers in the following 2 ways, which is the minimum number of participants that are allowed to be reported by *All of Us*. First, there was a drastic reduction in cohort size by 93.5%. However, because of the size of the overall dataset (N=372,397), we were still left with a large cohort (n=12,886), which, to the best of our knowledge, is the largest set of individuals to be analyzed for such a wide range of SDoH. Second, there were significant differences in the proportion of race, ethnicity, and age in the aforementioned cohort when compared to the overall *All of Us* population. Specifically, the cohort with valid answers had significantly more White, non-Hispanic, or older participants when compared to the overall cohort. This could potentially be because, once a participant has been enrolled, there is a 90-day delay in sending subsequent solicitations to complete surveys, a policy that is currently being reassessed due to its impact on missingness. Therefore, we had to correct this imbalance in demographic proportions by using IPW with the goal of identifying subtypes that were representative of the overall *All of Us* cohort.

#### Granularity

Because our goal was to use machine learning methods to identify SDoH subtypes, we encountered uneven granularity in the SDoH questions. Some questions were fine grained and highly correlated and, therefore, would cluster more strongly because of the nature of the granularity of the questions, not because of the SDoH mechanisms. To address this uneven granularity and make the results more interpretable, we used SDoH factors that had a coarser but more consistent level of granularity. We chose this approach because SDoH factors had already been defined; were understood by the expert panel, enabling high domain fidelity; and appeared to be at the right level of abstraction useful for clinical applications, such as referring a patient to the appropriate social services. However, because the use of coarse-grained variables loses information, future research could explore aggregating only those SDoH questions that are highly correlated while preserving the rest at the finer level of granularity and explore computational methods to merge SDoH questions into SDoH factors.

### Method: Scalability, Generalizability, and Extensibility

We designed the HIT analytical framework to be scalable, enabling its use for the growing size of the data in *All of Us*; generalizable across cohorts and conditions; and extensible for including additional methods as needed in the future. Testing the HIT framework on the *All of Us* dataset provided insights into the strengths and limitations of the framework and the *All of Us* workbench where the analysis was conducted.

#### Scalability

We used 3 types of code to conduct the analysis for both research questions. The first was automatically generated code to extract the cohort, produced by *All of Us* once a cohort was selected using the point-and-click interface. This code was adequately scalable and generalizable and so will not be discussed further. The second was customized code to extract specific parts of the data. For example, the analysis of co-occurrences required customized code in R (R Foundation for Statistical Computing) to plot the diagrams in Figures 4–6. As expected, these tasks required strong programming skills, but fortunately, we did not encounter any coding or execution problems using the R or Python (Python Software Foundation) programming languages. However, there were significant server issues that hampered our analysis. Although the workbench instructions stated that code running on the workbench for >2 weeks would be terminated and all intermediate results would be deleted, we frequently encountered our work disappearing at shorter intervals. These disruptions resulted in a higher consumption of the free server time credits resulting in fewer analyses that could be conducted. The third type of code was machine learning code that we had previously developed and disseminated on Comprehensive R Archive Network [78-80] to conduct the bipartite network analysis and the significance testing and visualize the network. As this code was designed to be generalizable and scalable, we did not encounter any issues in the execution of our code (in addition to the same server issues mentioned previously). Finally, the visualization of our networks worked as expected, and we used them to help interpret the patterns in the data.

#### Generalizability

Our code for the first 2 steps of the HIT framework is in Project Jupyter notebooks and has been used to analyze other cohorts that were filtered for age and previous conditions. For example, we extracted a cohort (n=4090) of participants with diabetes aged ≥65 years with complete data on 18 SDoH variables selected through consensus by 2 experienced health services researchers and guided by the Andersen behavioral model. The analysis [81,82] revealed 7 SDoH subtypes with statistically significant modularity compared with 100 random permutations of the data (*All of Us*=0.51; random mean 0.38 SD 0.0065; *z*=20; *P*<.001) and that were not only clinically meaningful but also significant in different degrees for the outcome. Our subsequent attempt at increasing the number of SDoH variables from 18 to 110 for participants with diabetes who had valid answers led to an extremely small cohort size (n=926; Multimedia Appendix 5) due to the missingness that we described previously. While this reduction resulted in our current strategy of analyzing all participants regardless of condition or age, these experiments demonstrate that our approach is generalizable to other subsets of the data.

#### Extensibility

The HIT model is designed to be extensible to include other methods. For example, the model could use other biclustering (eg, nonnegative matrix factorization [83]) and causal modeling methods and different types of classification (eg, deep learning [84]) and prediction methods (eg, subgroup-specific modeling [40]) to build the decision support system in the translational step (Figure 2). Furthermore, the model can integrate a wide range of datatypes to enable analysis of how each subtype is associated with them, resulting in a layered interpretation of the SDoH subtypes, as we have demonstrated. For example, as the percentage of participants who have genomic information increases (currently, >25% of our cohort have missing genomic information), our pipeline will be able to integrate such information into our analysis. Finally, the integration of different datatypes required a diverse team consisting of experts in machine learning, biostatistics, programming, clinical care, health services research, gerontology, and ethics to enable a 360° analysis and interpretation of the subtypes that was, therefore, aligned with the human-centered artificial intelligence approach [64-66]. Furthermore, the use of the workbench to share results through visualizations operationalized *team-centered informatics* [85] designed to facilitate multidisciplinary translational teams [86] working more effectively across disciplinary boundaries with the goal of analyzing subtypes and designing targeted interventions.

### Theory: Model Building and Translational Implications

The identification of SDoH subtypes has strong implications for model building in addition to translational applications. As shown in Figure 8, while the current classification of 5 SDoH domains has a hierarchical relationship with the SDoH factors, the data-driven clusters have a more complex association with the same SDoH factors. This reflects the complexity of how SDoH occur in the real world while at the same time being interpretable for purposes of translation.

Future studies should develop prediction models using the data-driven subtypes to determine whether they improve the accuracy of predicting adverse health outcomes when compared to models that do not use those subtypes. Because the subtypes were clinically interpretable, they could be used to build classification and prediction models and used with an interface to develop a clinical decision support system that helps triage patients to critical services. For example, the St Vincent’s House [87] in Galveston, Texas, United States, provides several services to address SDoH, including free walk-in clinical care, nurse practitioners with reduced insurance co-payments, English- and Spanish-speaking free mental health counseling, free dental health clinic, utility and rental assistance, case management, financial literacy, expanded food pantry, weekly free home delivery of pantry groceries, snack pack for people experiencing homelessness, free transportation to clinician’s appointments, immigration legal services, and spiritual counseling. Given the availability of this wide range of services in many communities across the United States, a decision support system could help classify an individual based on their SDoH profile into one or more of the subtypes and measure their risk of an adverse health outcome. Such information could be used by clinicians to collaboratively explore solutions with the patient to consider one or more of such local services based on the membership strength to a subtype and the associated risk (Figure 2, step 3). At a population level, understanding health risks associated with clusters may assist institutions and organizations in developing more effective prevention programs.

### Notebooks for *All of Us* Community Use

Because the missingness in SDoH variables is expected to decrease, their characterization and subtyping will need to be repeated and verified for different cohorts. Therefore, we have made the following 2 sets of code available for general use by the *All of Us* researcher community (accessible after creating a free account on *All of Us* and completing the required training).

#### SDoH Valid Answer Tracker

This set of notebooks generates four plots that can be used by other researchers on *All of Us* to characterize any cohort: (1) valid response plot to show how many participants have data with valid responses and colored by SDoH domain, (2) Venn diagram showing how many participants have valid responses for all questions within each survey, and (3) frequency distribution plot showing co-occurrence of SDoH across the selected cohort. This set of tools should enable researchers to characterize SDoH across different cohorts to help determine methods that are appropriate to adjust for missingness in those cohorts.

#### SDoH Subtyper

This set of notebooks can be used to conduct the following analyses: (1) bicluster modularity of a cohort with the 18 SDoH factors to identify the number and members of biclusters and the measure *Q* representing the quality of the biclustering, (2) visualization of the bipartite network, and (3) significance of the network with respect to null models.

### Limitations

This study has 2 main limitations. The first emerges from the large amount of missingness in the survey data, precluding the use of imputation methods that assume a random distribution of missingness. Therefore, we could use only complete data, which led to a large drop in cohort size and which also introduced a bias in the demographics requiring a rebalancing through IPW. While such rebalancing is typically done for large datasets, the IPW method requires judgment to decide which variables to include in the model and, therefore, could have introduced additional unknown biases. Therefore, the model should be refined to determine which variables to include in the regression models that estimate the IPW-generated weights. However, because the clustering was similar between the unweighted and IPW-weighted networks, we believe that the current subtypes are stable and meaningful and represent the demographic composition of the full *All of Us* dataset, but this needs to be verified by redoing the analysis as the data become more complete. The limitation of missingness in the surveys is expected to be addressed as *All of Us* has recently removed the requirement of waiting 90 days before a subsequent survey is given to an enrollee in the program, potentially reducing the degree of missingness. The second limitation is due to the high computational cost of empirically determining the significance of the biclustering. As such analysis is computationally expensive and time-consuming, it limited the experiments we could do to test different cohorts and models. Therefore, we look forward to the *All of Us* workbench providing the ability to run batch processes more efficiently and uninterruptedly for extended periods (exceeding the current time window), which together could help alleviate this computational hurdle in the future.

### Conclusions

How SDoH impact health is a complex phenomenon involving many interconnected social, biological, and environmental factors that have yet to be fully elucidated. While this phenomenon has been studied for >30 years, the analyses have been hampered by the lack of large cohorts representing diverse populations with a wide range of SDoH variables measured, multiple datatypes, and easy access by researchers. *All of Us* provides an unprecedented opportunity to directly address these limitations with the goal of doing justice to early conceptual models such as the social gradient and the Dahlgren-Whitehead model, both of which drew international attention to the complex ways in which individual and contextual SDoH factors impact health. The *All of Us* dataset is also timely because of the extensive health disparities that were revealed during the pandemic, which highlighted the critical need to address SDoH in the public and policy realms. However, because *All of Us* is still rapidly evolving to meet its target of 1 million participants or more, we conducted a systematic characterization of SDoH variables in *All of Us* and used the results to guide the analysis of SDoH subtypes. The identified subtypes, along with their risks, could be used to design data-informed interventions, resource-planning strategies, and public health policies aimed at reducing the risks of adverse health outcomes. However, careful consideration would be required to ensure that the identification of high-risk subtypes is not used in a way that stigmatizes subpopulations.

Our first goal of characterizing the data revealed the nature of the missingness in SDoH and the uneven granularity in the SDoH questions. Both of these results led us to select the IPW method to address the missingness and to analyze subtypes using SDoH factors to address the uneven granularity. Our second goal of identifying SDoH subtypes led not only to statistically significant biclusteredness but also to their statistically significant replication and meaningful domain interpretations. These results set the stage for further investigations to build and evaluate classification and prediction models for designing decision support systems that alert clinicians of specific risks that their patients face due to a combination of SDoH factors. Furthermore, the SDoH subtypes could be used to design public health care policies that are multifactorial and need based, enabling more targeted interventions compared to policies that are based on a few sociodemographic factors such as race and income.

The results also led to the design, use, and dissemination of general-purpose tools currently available on *All of Us* for other researchers, which will be useful to reanalyze the *All of Us* dataset as it grows over the next few years to directly address the high rate of missingness. These collaborative advances should position *All of Us* to revolutionize research for analyzing complex phenomena such as how SDoH impact health and beyond, with the goal of enabling a more equitable future that all of us deserve.

## Appendix

Multimedia Appendix 1 Description of bipartite network analysis.

Multimedia Appendix 2 Systematized Medical Nomenclature of Medicine codes related to social determinants of health and their use in the electronic health records of participants in All of Us.

Multimedia Appendix 3 Data conversion. A total of 4 *All of Us* surveys (column 2) contained 110 social determinants of health questions (column 3), which were abbreviated, negatively phrased (shown bolded), and reversed coded (shown in red; column 3); categorized into the 5 Healthy People 2030 domains (column 4 and shown by the 5 colors); and further categorized (boxes) by the expert panel into 18 factors (column 5; Delayed medical care was used as an outcome).

Multimedia Appendix 4 Inverse probability weighting.

Multimedia Appendix 5 Condition-specific cohort extraction for type 2 diabetes, breast cancer, and coronary artery disease, and inclusion and exclusion criteria for selecting 3 condition-specific cohorts.

## Abbreviations

EHR: electronic health record
ER: emergency room
HIT: heterogenization, integration, and translation
HP-30: Healthy People 2030
IPW: inverse probability weighting
OR: odds ratio
RI: Rand index
SDoH: social determinants of health
SNOMED: Systematized Medical Nomenclature of Medicine

## Data Availability

The datasets analyzed in this study are publicly available from the *All of Us* program after registering for access [88].
