# Supplementary material for: Subtyping Social Determinants of Health in the "All of Us" Program: Network Analysis and Visualization Study
Source: J Med Internet Res. 2025 Feb 11;27:e48775. doi: 10.2196/48775 (PMC11862773; doi:10.2196/48775)
Supplement: Multimedia Appendix 1 [file jmir_v27i1e48775_app1.docx]

**Multimedia Appendix 1.** Description of Bipartite Network Analysis.

A network consists of nodes and edges; nodes represent one or more types of entities (e.g., participants or SDoH), and edges between the nodes represent a specific relationship between the entities. Figure 1A shows a unipartite network where nodes are the same type (typically used to analyze co-occurrence of comorbidities [1]). In contrast, Figure 1B shows a bipartite network where nodes are of two types, and edges exist only between different types such as between participants (circles) and SDoH (triangles). Bipartite network analysis takes as input any dataset such as *All of Us* participants and their SDoH, and automatically outputs a quantitative and visual description of biclusters (containing both participant subgroups, and their frequently co-occurring SDoH). The quantitative output provides the number, size, and statistical significance of the biclusters [2-4], and the visual output displays the quantitative information of the biclusters through a network visualization [5-7]. Bipartite network analysis therefore enables (1) the automatic identification of biclusters and their significance, and (2) the visualization of the biclusters critical for their clinical interpretability including labeling the subtypes, inferring potential mechanisms that precipitate adverse outcomes in each subtype, and designing targeted interventions to prevent them. Furthermore, the characteristics (e.g., outcomes and covariates) of participants in a subtype can be used to measure the risk of a subtype for an adverse outcome when compared to a reference group (e.g., a control group or another subtype), and therefore enables the integration of multiple data types. Finally, the biclusters can be used to develop classifiers for classifying a new participant into one or more of the subtypes, and developing a predictive model that uses those subtype membership for measuring the risk of an adverse outcome for that new participant.

**
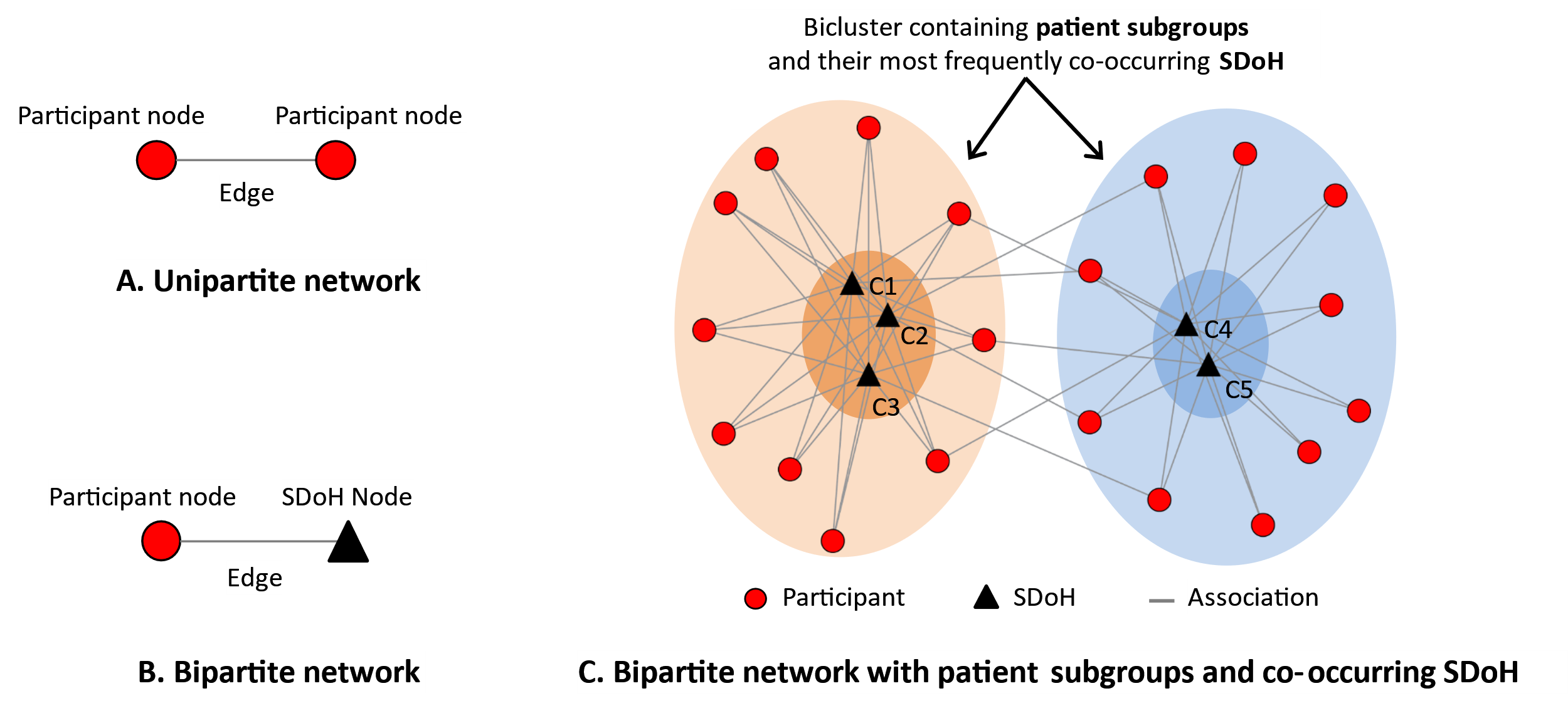
**

**Figure S1.** The distinction between a unipartite network (A), a bipartite network (B), and how the latter can be used to identify biclusters of participants and their most frequently co-occurring SDoH (C).

# **References**

1. Folino, F., C. Pizzuti, and M. Ventura, *A comorbidity network approach to predict disease risk*, in *Proceedings of the First international conference on Information technology in bio- and medical informatics*. 2010, Springer-Verlag: Bilbao, Spain. p. 102-109.

2. Treviño, S., et al., *Fast and accurate determination of modularity and its effect size.* Journal of Statistical Mechanics: Theory and Experiment, 2015. **2015**(2): p. P02003.

3. Chauhan, R., et al., *Reconstruction and topological characterization of the sigma factor regulatory network of Mycobacterium tuberculosis.* Nat Commun, 2016. **7**: p. 11062.

4. Bhavnani, S.K., et al., *How High-Risk Comorbidities Co-Occur in Readmitted Patients With Hip Fracture: Big Data Visual Analytical Approach.* JMIR Med Inform, 2020. **8**(10): p. e13567.

5. Fruchterman, T. and E. Reingold, *Graph Drawing by Force-Directed Placement.* Software – Practice & Experience, 1991. **21**(11): p. 1129–1164.

6. Dang, B., et al., *ExplodeLayout: Enhancing the Comprehension of Large and Dense Networks*, in *AMIA Jt Summits Transl Sci Proc.* . 2016.

7. Bhavnani, S.K., et al., *Enabling Comprehension of Patient Subgroups and Characteristics in Large Bipartite Networks: Implications for Precision Medicine.* AMIA Jt Summits Transl Sci Proc, 2017: p. 21-29.
