# Supplementary material for: Subtyping Social Determinants of Health in the "All of Us" Program: Network Analysis and Visualization Study
Source: J Med Internet Res. 2025 Feb 11;27:e48775. doi: 10.2196/48775 (PMC11862773; doi:10.2196/48775)
Supplement: Multimedia Appendix 2 [file jmir_v27i1e48775_app2.docx]

**Multimedia Appendix 2.** SNOMED Codes Related to SDoH, and their Use in the Electronic Health Records of Participants in *All of Us.*

**
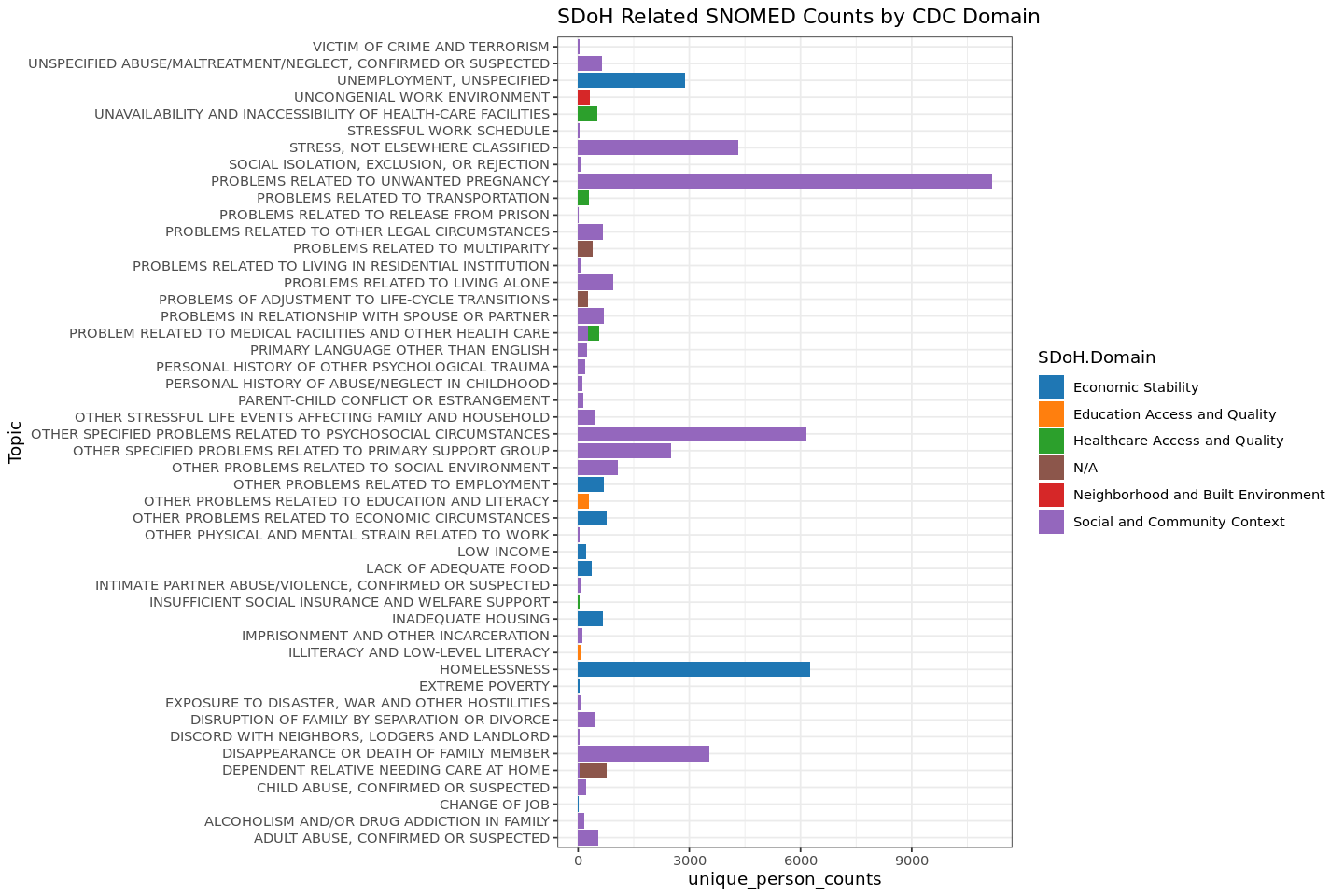
**
