# Supplementary material for: Subtyping Social Determinants of Health in the "All of Us" Program: Network Analysis and Visualization Study
Source: J Med Internet Res. 2025 Feb 11;27:e48775. doi: 10.2196/48775 (PMC11862773; doi:10.2196/48775)
Supplement: Multimedia Appendix 3 [file jmir_v27i1e48775_app3.docx]

**Multimedia Appendix 3.** Data Conversion.

Four *All of Us* surveys (Column-2), contained 110 SDoH questions (Column-3), that were abbreviated, negatively phrased (shown bolded) and reversed coded (shown in red) (Column-3), categorized into the five *HP-30* domains (Column-4 and shown by the five colors), and further categorized (boxes) by the expert panel into 18 factors (Column-5; *Delayed Medical Care* was used as an outcome).
