# Supplementary material for: Subtyping Social Determinants of Health in the "All of Us" Program: Network Analysis and Visualization Study
Source: J Med Internet Res. 2025 Feb 11;27:e48775. doi: 10.2196/48775 (PMC11862773; doi:10.2196/48775)
Supplement: Multimedia Appendix 4 [file jmir_v27i1e48775_app4.docx]

**Multimedia Appendix 4.** Inverse Probability Weighting (IPW).

We found significant differences in the demographic proportions between our cohort (n=12,913) consisting of participants with valid answers for all 110 SDoH questions, and the total *All of Us* data. To adjust for potential sample selection bias, we calculated inverse probability weights (IPW) using the *ipwpoint* function in the R package *ipw*.^66^ This function uses a logistic regression model to estimate the predicted probability of having valid responses on all SDoH variables based on age, sex, race, ethnicity, being born in the United States, currently employed, having a college degree or higher, health insurance, owning a home, and being married. We stabilized the weights according to the observed probability of being in our cohort. The resulting IPW weights were used as weights for the edges in the bipartite network.
