# Supplementary material for: Subtyping Social Determinants of Health in the "All of Us" Program: Network Analysis and Visualization Study
Source: J Med Internet Res. 2025 Feb 11;27:e48775. doi: 10.2196/48775 (PMC11862773; doi:10.2196/48775)
Supplement: Multimedia Appendix 5 [file jmir_v27i1e48775_app5.docx]

**Multimedia Appendix 5.** Condition-specific cohort extraction for type II diabetes (T2DM), breast cancer, and coronary artery disease (CAD).


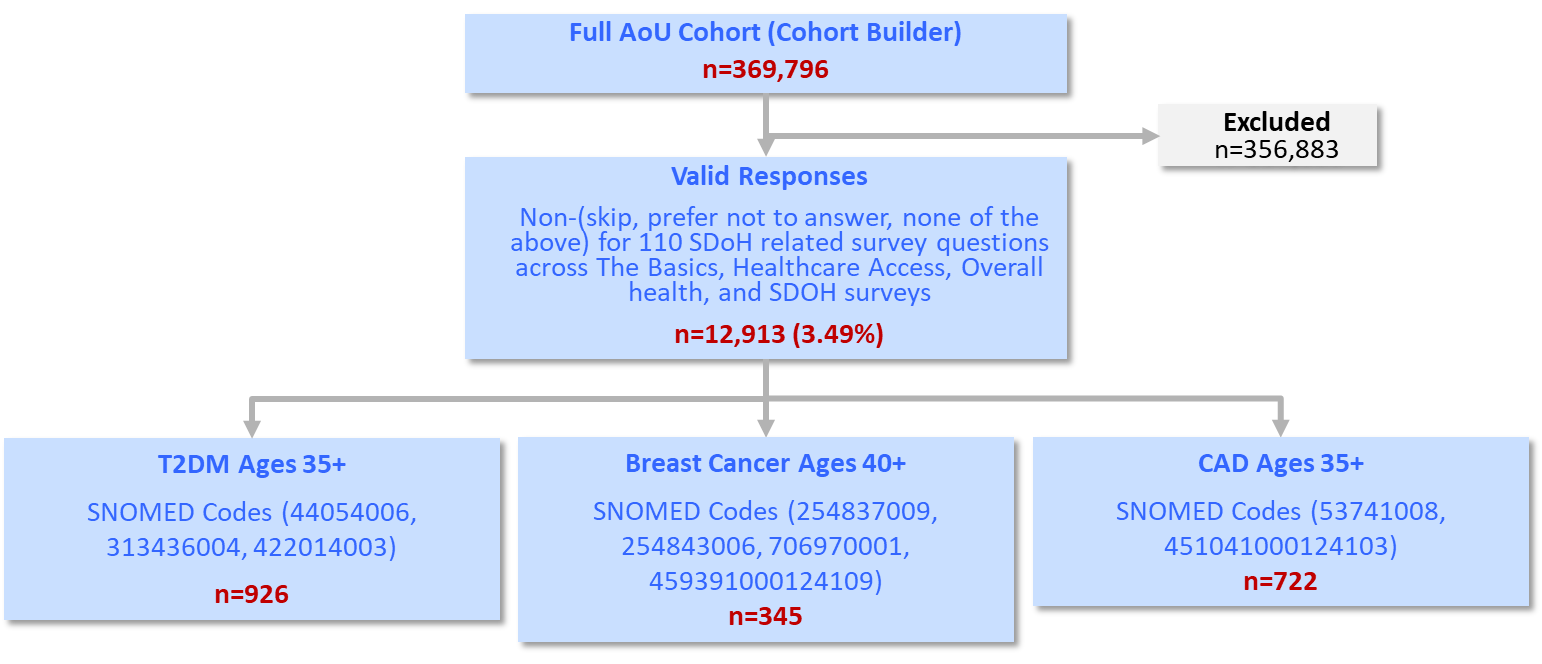


**Figure S1.** Inclusion and exclusion criteria for selecting three condition-specific cohorts.
